# Isolation and characterization of algicidal bacteria from freshwater aquatic environments in China

**DOI:** 10.3389/fmicb.2023.1156291

**Published:** 2023-03-09

**Authors:** Sanguo Ren, Yuanpei Jin, Jianan Ma, Ningning Zheng, Jie Zhang, Xingyu Peng, Bo Xie

**Affiliations:** School of Life Sciences, Hubei Key Laboratory of Genetic Regulation and Integrative Biology, Central China Normal University, Wuhan, China

**Keywords:** algicidal bacteria, bacteria-algae interaction, harmful algal bloom, phylogenetic diversity, algal biotechnology

## Abstract

Algicidal bacteria can inhibit the growth of algae or lyse algal cells, thus playing roles in shaping aquatic microbial communities and maintaining the functions of aquatic ecosystems. Nevertheless, our understanding of their diversities and distributions remains limited. In this study, we collected water samples from 17 freshwater sites in 14 cities in China and screened a total of 77 algicidal bacterial strains using several prokaryotic cyanobacteria and eukaryotic algae as target strains. According to their target-specificities, these strains were classified into three subgroups, cyanobacterial algicidal bacteria, algal algicidal bacteria, and broad-target algicidal bacteria, each displaying distinctive compositions and geographical distribution patterns. They are assigned to Proteobacteria, Firmicutes, Actinobacteria, and Bacteroidetes bacterial phyla, of which *Pseudomonas* and *Bacillus* are the most abundant gram-negative and gram-positive genus, respectively. A number of bacterial strains, such as *Inhella inkyongensis* and *Massilia eburnean*, are suggested as new algicidal bacteria. The diverse taxonomies, algal-inhibiting abilities and distributions of these isolates have suggested that there are rich algicidal bacterial resources in these aquatic environments. Our results provide new microbial resources for algal-bacterial interaction studies, and shed new insights into how algicidal bacteria can be used in the control of harmful algal blooms, as well as in algal biotechnology.

## Introduction

Aquatic microbial communities contain numerous autotrophic and heterotrophic microbial species, which are essential for global primary production and biogeochemical cycles (Field et al., 1998). Extensive studies have revealed the key members within these communities, such as phytoplankton and other bacteria, can form either beneficial or negative interactions that can have substantial effects on the growths and functions of both partners (Cole, 1982; Kim et al., 2009; Amin et al., 2012; Xie et al., 2013; Cooper et al., 2019). These phytoplankton-bacteria interactions are critical ecological relationships in aquatic systems, and play roles in shaping aquatic microbial communities and maintaining the functions of water ecosystems (Seymour et al., 2017).

Among these bacteria, algicidal bacteria are the group that can greatly inhibit the growth of algae or lyse algal cells (Meyer et al., 2017; Coyne et al., 2022). These bacteria have been extensively isolated from various environments such as lakes, seas and lands in past decades. Some algicidal bacteria were associated with the development and decline of algal blooms (Mayali and Azam, 2004). They are taxonomic diverse and may specifically inhibit certain prokaryotic cyanobacteria or eukaryotic algae. It has been indicated that most of the isolated algicidal bacteria against algae are from Bacteroidetes and Proteobacteria, while those against cyanobacteria may be widely found in Firmicutes, Proteobacteria and Actinobacteria (Meyer et al., 2017; Yang et al., 2020; Coyne et al., 2022). Interspecies interactions between these bacteria and the target phytoplankton may be cell–cell contact dependent or independent, and involve a wide range of algicides such as alkaloids, amino acid derivatives, peptides, or other metabolites (Meyer et al., 2017). For example, Marine bacterium *Cytophaga* sp. J18/M01 can kill marine phytoplankton such as *Chattonella antiqua* and *Skeletonema costatum* by direct attack (Imai et al., 1993). *Aeromonas* sp. GLY2107 produces two algicidal compounds, 3-benzyl-piperazine-2,5-dione, and 3-methylindole, which can kill the bloom-forming cyanobacterium *Microcystis aeruginosa* (Guo et al., 2016). *Deinococcus xianganensis* Y35 produces algicidal compound deinoxanthin, a red pigment, which is toxic to alga *Alexandrium tamarense* (Li et al., 2015). Our recent isolated bacterium, *Paenibacillus polymyxa* MEZ6, can rapidly kill several eukaryotic algal strains, and certain glycoside hydrolases and antimicrobial secondary metabolites are suggested to play roles in its algicidal processes (Zhao et al., 2022). Complex relationships between bacteria and phytoplankton, like multiple-species algal-inhibiting interactions and switchable interaction from mutualism to antagonism, have also been reported (Seyedsayamdost et al., 2011; Krespach et al., 2020). However, our current understanding of the diversities, distributions and target-specificities of algicidal bacteria is still limited.

With the strong inhibitory activities, algicidal bacteria have been of great interest in harmful algae blooms (HABs) control and algal biotechnology in recent years (Sun et al., 2018; Wang et al., 2020; Coyne et al., 2022). HABs are formed in eutrophic waters all over the world, representing a serious environmental problem in recent years (Harke et al., 2016; Ho et al., 2019; Huo et al., 2021). Certain toxic species (e.g., *M. aeruginosa*) can have a significant impact on human and animal health (Huisman et al., 2018). Previous studies suggested the other bacteria can play roles in the formation of HABs (Zhou et al., 2014; Seymour et al., 2017; Shao et al., 2020), and the use of algicidal bacteria can be a potentially efficient and eco-friendly approach in HABs control (Imai et al., 2021; Coyne et al., 2022). In fact, promising effects were observed in the evaluations of algicidal bacteria. For example, algicidal bacterium *Pseudomonas fluorescens* HYK0210-SK09 and its immobilized cells can effectively mitigate *Stephanodiscus hantzschii* bloom in mesocosm experiments (Jung et al., 2010, 2013). Cell-free filtrates from bacterium *Shewanella* sp. IRI-160 can inhibit the natural dinoflagellate blooms in microcosms (Tilney et al., 2014). In China, with the water eutrophication in past decades, HABs were increasingly detected in both freshwater bodies (e.g., Chao Lake, Dianchi Lake, and Erhai Lake) and seas (e.g., Bohai Sea and South China Sea) (Huo et al., 2021; Guan et al., 2022). *Microcystis* species are the most frequent taxa found in cyanobacterial blooms in China (Huo et al., 2021). Microbial agents consisting of either one species or multi-species of algicidal bacteria have been evaluated in the control of HABs in China (Ye et al., 2022). For example, bacterium *Bacillus cereus* CZBC1 can effectively control cyanobacterial bloom in shrimp culture systems (Xu et al., 2019). The multi-species algicidal bacteria enriched from eutrophication water have shown joint effects in the control of harmful algae (He et al., 2021). Additionally, in the algae-based biosynthetic process, algicidal bacteria can be used to disrupt algal cells, which may facilitate the recovery of bioproducts (Wang et al., 2020). However, the activities of algicidal bacteria can be greatly affected by environmental conditions and their actual large-scale field applications are still limited (Noh et al., 2017; Coyne et al., 2022; Ye et al., 2022). Systematic isolations and identifications of algicidal bacteria are needed to improve their applications in HABs control and algal biotechnology.

In the present study, we collected water samples from 17 freshwater sites in China, and screened for algicidal bacteria using several cyanobacteria and algae as target strains. Bacterial phylogenetic diversities were analyzed and their algal-inhibiting abilities and target-specificities were compared. Our results may provide new microbial resources for algal-bacterial interaction studies and improve the applications of algicidal bacteria in HABs control and algal biotechnology.

## Materials and methods

### Lake samples and bacterial isolation

All the water samples were collected at 2020–2021 from 17 freshwater environments, including lakes, rivers and pools, in 14 cities of China (Supplementary Table S1). At each site, 0.5-m depth water samples were collected in sterilized bottles and kept in ice. In the sites in Wuhan city, the samples were immediately transported back to the laboratory for bacterial isolation. In the sites outside of Wuhan city, the samples were shipped with ice packs to the laboratory within 2 days.

For bacterial isolation, water sample was directly spread on R2A (Reasoner and Geldreich, 1985), 1/2TY (Beringer, 1974), or M9 (Miller, 1972) agar and incubated at 28°C for 1 week. Representative bacterial colonies based on colony morphology observation were selected and streaked for purification. Each bacterial isolate was then separately co-inoculated with the target strains for algicidal activity tests.

### Algal and cyanobacterial strains

Several strains of cyanobacteria and algae were used to test bacterial algicidal activities. *M. aeruginosa* FACHB-524 and *M. aeruginosa* FACHB-927 were seleced since *Microcystis* species are the most frequently detected bloom-forming cyanobacteria in Chinese freshwater systems (Huo et al., 2021). *Chlamydomonas reinhardtii* 21gr (CC1690) and *Chlorella* sp. FACHB-1463 were included as these algal taxa can be used for the productions of biomass or high-value bioproducts (Liang et al., 2019). Two other cyanobacterial strains, *Anabaena* 7,120 and *Synechocystis* sp. PCC6803, were also included for a better understanding of bacterial target-specificity. *C. reinhardtii* 21gr was from Chlamydomonas Resource Center,^1^ and the other strains were from Freshwater Algae Culture Collection at the Institute of Hydrobiology, Wuhan, China (FACHB).^2^ Algal and cyanobacterial strains were grown in Tris-acetone-phosphate (TAP) (Gorman and Levine, 1965) and BG-11media (Rippka et al., 1979), respectively, at 25°C under a continuous light condition (120 μmol photons m^−2^ s^−1^).

### Co-inoculation and algicidal rate

Briefly, bacterial cells were washed twice and re-suspended in TAP or BG-11 medium before inoculating them into the algal or cyanobacterial cultures, respectively. The target cells were adjusted to 10^6^–10^7^ cell/ml. In the initial screen, the inoculum ratio of bacterial cells: algal cells was approximately 100:1 and different inoculum ratios can be used when required. For the co-inoculations using bacterial lysate, 2-day-old bacterial (in R2A or 1/2TY medium) was centrifuged at 10,000 g for 6 min and cells were re-suspended in TAP or BG-11 solution. Cells were sonicated and the lysate was centrifuged to remove cell debris before its supplementation into the target culture (5% vol/vol). To examine whether a cell–cell contact is required for algicidal activity, the 12-well Tissue Culture Plate Insert (Labselect, China) was used for the co-inoculations, in which bacteria and the target cells were separated with a 0.1 μm polycarbonate filter.

Algicidal rates were determined 7 days after inoculation (DAI) using the formula, (1-Chlorophyll_+bacteria_/Chlorophyll_control_) × 100%, where Chlorophyll_+bacteria_ is the chlorophyll content of the co-culture, and Chlorophyll_control_ is the chlorophyll content of alga/cyanobacterium alone. Chlorophyll *a* for cyanobacteria and total chlorophyll contents for algae were determined as described previously (Minocha et al., 2009). All co-inoculations were performed at least with 3 independent replications. For multiple group comparisons, one-way ANOVA followed by Duncan post-hoc test was used with SPSS 19 software (IBM, United States). In this study, only the bacterial strains with an algicidal rate ≥ 60% against at least one target algal/cyanobacterial strain were selected for the downstream studies.

### Phylogenetic tree

Bacterial 16S rRNA gene was amplified using universal primers 27F and 1492R (Frank et al., 2008), and the sequence was searched in EzBioCloud databases to find the closest 16S rRNA gene. Multiple 16S rRNA gene sequences were aligned using CLUSTALW and the phylogenetic trees were constructed using neighbor-joining method with MEGA X software (Kumar et al., 2018).

## Results

### Samples and bacteria isolations

To investigate the distribution and diversity of algicidal bacteria in freshwater aquatic environments in China, we collected water samples from 17 freshwater sites, including lakes, rivers and pools, in 14 Chinese cities which are mainly located in central and southern China (Supplementary Table S1). For example, Wuhan city is located in central China, and has more than 100 urban lakes. Three sampling sites were selected in Wuhan city, including Nan Lake, Tangxun Lake and our Central China Normal University (CCNU) campus, which are convenient for sampling and transportation. The sites in Yunnan province (Yilong Lake and Erhai Lake) and Hainan province (Meilun River) are located in southern China. Some lakes (e.g., Nan Lake, Chao Lake and Yilong lake) have experienced cyanobacteria bloom in recent years. These geographically distinct samples may harbor different microbial communities and algicidal bacterial resources.

More than 1,500 bacterial strains were isolated from these samples and their algal-inhibiting abilities were tested using 6 target algal and cyanobacterial strains, according to Materials and Methods. Finally, a total of 77 bacterial strains with a strong algae-inhibiting ability (algicidal rate ≥ 60%) against at least one target cyanobacterial or algal strain were obtained (Supplementary Table S2). All these algicidal bacterial isolates implied there are rich microbial resources in our samples for the antagonistic interaction studies between phytoplankton and other bacteria. They were used for the downstream studies of algicidal ability, target-specificity, and phylogenetic analysis.

### Algicidal abilities and specificities of bacterial isolates

The algal-inhibiting abilities of algicidal bacterial can be highly specific to the targets. We found there are about 57 and 65% of the 77 isolates showing high algicidal abilities against cyanobacteria and algae, respectively. About 23% of these isolates can broadly inhibit the growth of both cyanobacteria and algae (Supplementary Table S2). Thus, we classified these strains into three subgroups (Supplementary Table S2), cyanobacterial algicidal bacteria (algicidal rate ≥ 60% only for the tested prokaryotic cyanobacterial strains), algal algicidal bacteria (algicidal rate ≥ 60% only for the tested eukaryotic algal strains) and the broad-target algicidal bacteria for both cyanobacteria and algae (algicidal rate ≥ 60% for both cyanobacterial and algal strains).

Interestingly, about half of the strains (36 out of 77) were effective on only one target species, and almost no strains could strongly inhibit all the tested algal species. As shown in Table 1, while 44 isolates (e.g., MERWZ-1, MERYL1-3) can strongly inhibit the growth of *C. reinhardtii* 21gr, only 6 (e.g., MERTX3-1) are effective on *Anabaena* sp. PCC 7120. Algicidal bacterial isolate numbers for *M. aeruginosa* and *Synechocystis* sp. PCC 6803 are 28 and 23, respectively. These results indicated target-specificities and algicidal abilities can be highly different among our isolates.

**Table 1:** Summary of algicidal bacterial isolates.

Target algae/cyanobacteria	Number of algicidal isolates	Closest taxonomy
Cyanobacteria		
*Anabaena* sp. PCC 7120	6	*Bacillus velezensis*, Inhella inkyongensis*, *Serratia nematodiphila*, *Paucibacter aquatile*, *Chitinimonas viridis*
*Synechocystis* sp. PCC 6803	23	*Microbacterium oxydans*, *Micrococcus endophyticus*, Flectobacillus roseus*, *Flavobacterium chryseum*, *Sphingobacterium mucilaginosum*, *Bacillus altitudinis*, *Exiguobacterium artemiae*, *Exiguobacterium undae*, Asticcacaulis excentricus*, Allorhizobium pseudoryzae*, *Rhizobium wuzhouense*, *Hydrogenophaga laconesensis*, Roseateles terrae*, *Serratia nematodiphila*, *Serratia marcescens*, *Microbacterium arabinogalactanolyticum*, *Streptomyces roseolus*, *Paucibacter aquatile*, *Chitinimonas viridis*, *Chromobacterium rhizoryzae*
*Microcystis aeruginosa* FACHB-927	18	*Bacillus velezensis*, *Lysinibacillus fusiformis*, *Sphingomonas echinoides*, *Achromobacter marplatensis*, *Hydrogenophaga flava*, *Paucibacter aquatile*, Roseateles terrae*, Microvirga calopogonii*, *Serratia nematodiphila*, *Serratia marcescens*, *Achromobacter ruhlandii*, *Chitinimonas viridis*, *Chromobacterium rhizoryzae*
*Microcystis aeruginosa* FACHB-524	25	*Achromobacter marplatensis*, *Achromobacter ruhlandii*, *Bacillus paralicheniformis*, *Bacillus velezensis*, *Chitinimonas viridis*, *Chromobacterium rhizoryzae*, *Hydrogenophaga flava*, *Hydrogenophaga taeniospiralis*, Kinneretia asaccharophila*, *Lysinibacillus fusiformis*, Oleisolibacter albus*, *Paucibacter aquatile*, *Serratia marcescens*, *Serratia nematodiphila*, *Sphingomonas echinoides*
Algae		
*Chlamydomonas reinhardtii* 21gr	44	*Achromobacter marplatensis*, *Achromobacter ruhlandii*, *Acidovorax* RCCC_s, *Acinetobacter lwoffii*, *Acinetobacter* NHRN_s, *Aeromonas caviae*, *Bacillus altitudinis*, *Bacillus cereus*, *Bacillus velezensis*, Chromobacterium rhizoryzae*, *Delftia tsuruhatensis*, Flectobacillus roseus*, *Hydrogenophaga taeniospiralis*, Kinneretia asaccharophila*, Massilia eburnean*, *Microbacterium arabinogalactanolyticum*, *Paenibacillus polymyxa*, *Paucibacter aquatile*, *Pseudomonas* CP032618_s, *Pseudomonas hunanensis*, *Pseudomonas japonica*, *Pseudomonas mosselii*, *Pseudomonas otitidis*, *Pseudomonas* QJRX_s MB-090714, *Pseudomonas soli*, Pseudoxanthomonas arseniciresistens*, *Sphingomonas echinoides*, *Streptomyces roseolus*, *Streptomyces violascens*, Vogesella urethralis*
*Chlorella* sp.FACHB-1463	31	*Aeromonas caviae*, *Aeromonas enteropelogenes*, *Bacillus albus*, *Bacillus cereus*, *Chitinimonas viridis*, *Chromobacterium rhizoryzae*, *Delftia tsuruhatensis*, Flectobacillus roseus*, *Hydrogenophaga taeniospiralis*, Kinneretia asaccharophila*, *Paenibacillus polymyxa*, *Paucibacter aquatile*, *Pseudomonas hunanensis*, *Pseudomonas japonica*, *Pseudomonas mosselii*, *Pseudomonas piscium*, *Pseudomonas soli*, *Sphingomonas echinoides*, *Streptomyces violascens*

* Rare algicidal bacteria in this genus were reported.

### Bacterial diversities

**Figure 1:**
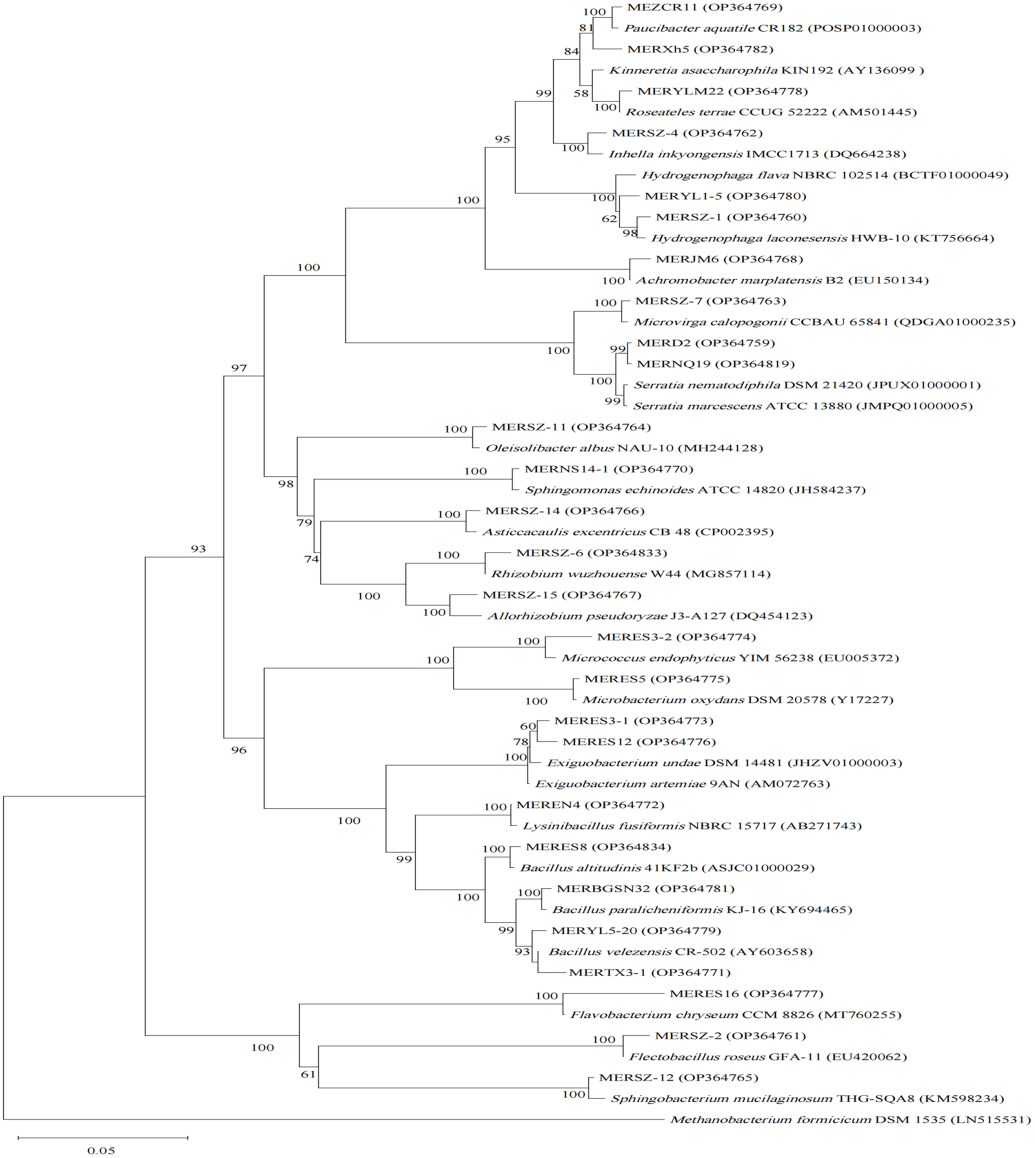
Neighbor-joining phylogenetic tree of 16S rRNA gene sequences of the algicidal bacterial isolates for cyanobacteria, and their closest relatives. Accession numbers of the isolates (GenBank) and their relatives (EZbiocloud) are shown in parentheses. *Methanobacterium formicicum* DSM 1535 was used as outgroup. Bootstrap values ≥50% (Bootstrap = 1,000) are shown at the branch nodes. Bar, 0.05 substitutions per nucleotide position.

**Figure 2:**
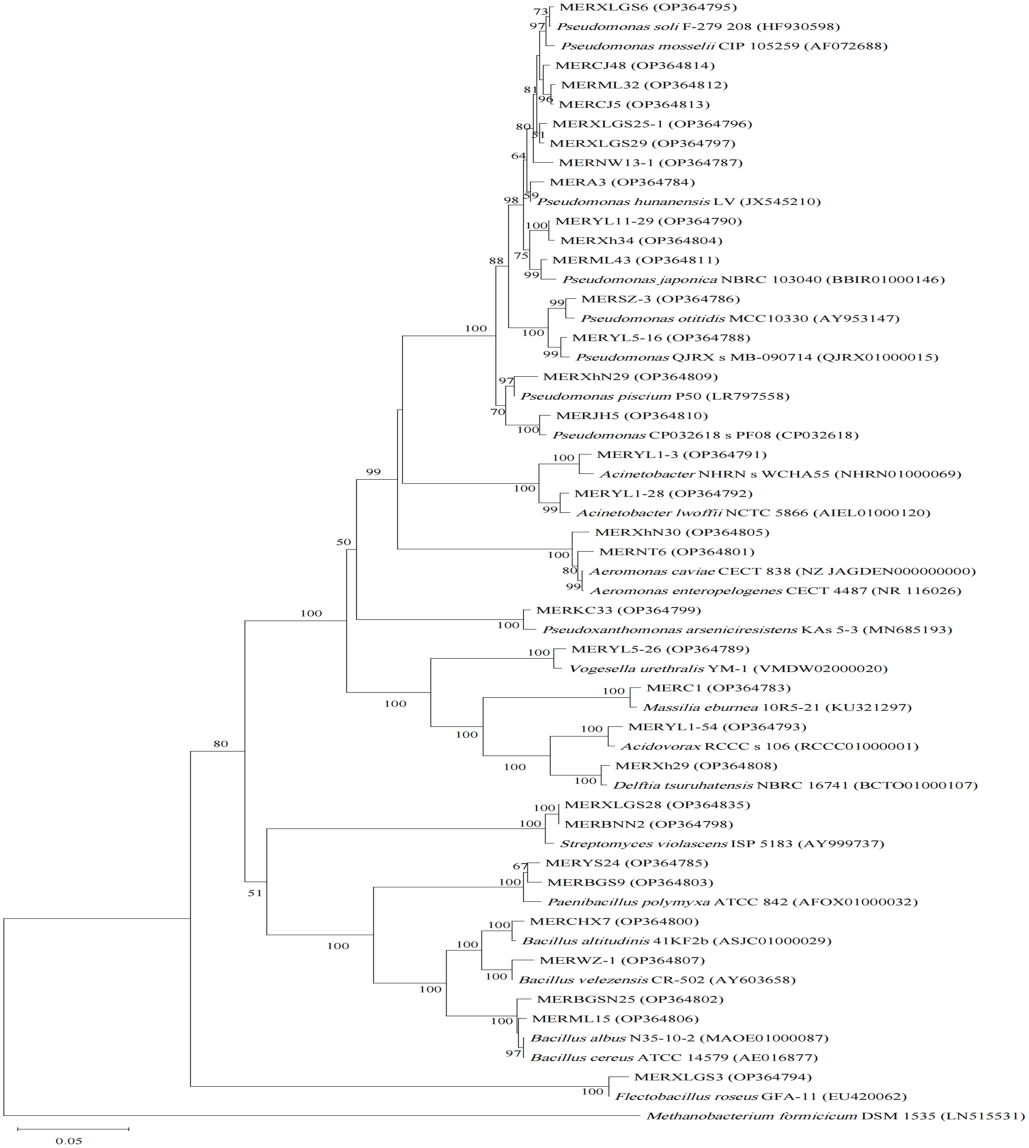
Neighbor-joining phylogenetic tree of 16S rRNA gene sequences of the algicidal bacterial isolates for algae, and their closest relatives. Accession numbers of the isolates (GenBank) and their relatives (EZbiocloud) are shown in parentheses. *Methanobacterium formicicum* DSM 1535 was used as outgroup. Bootstrap values ≥50% (Bootstrap = 1,000) are shown at the branch nodes. Bar, 0.05 substitutions per nucleotide position.

**Figure 3:**
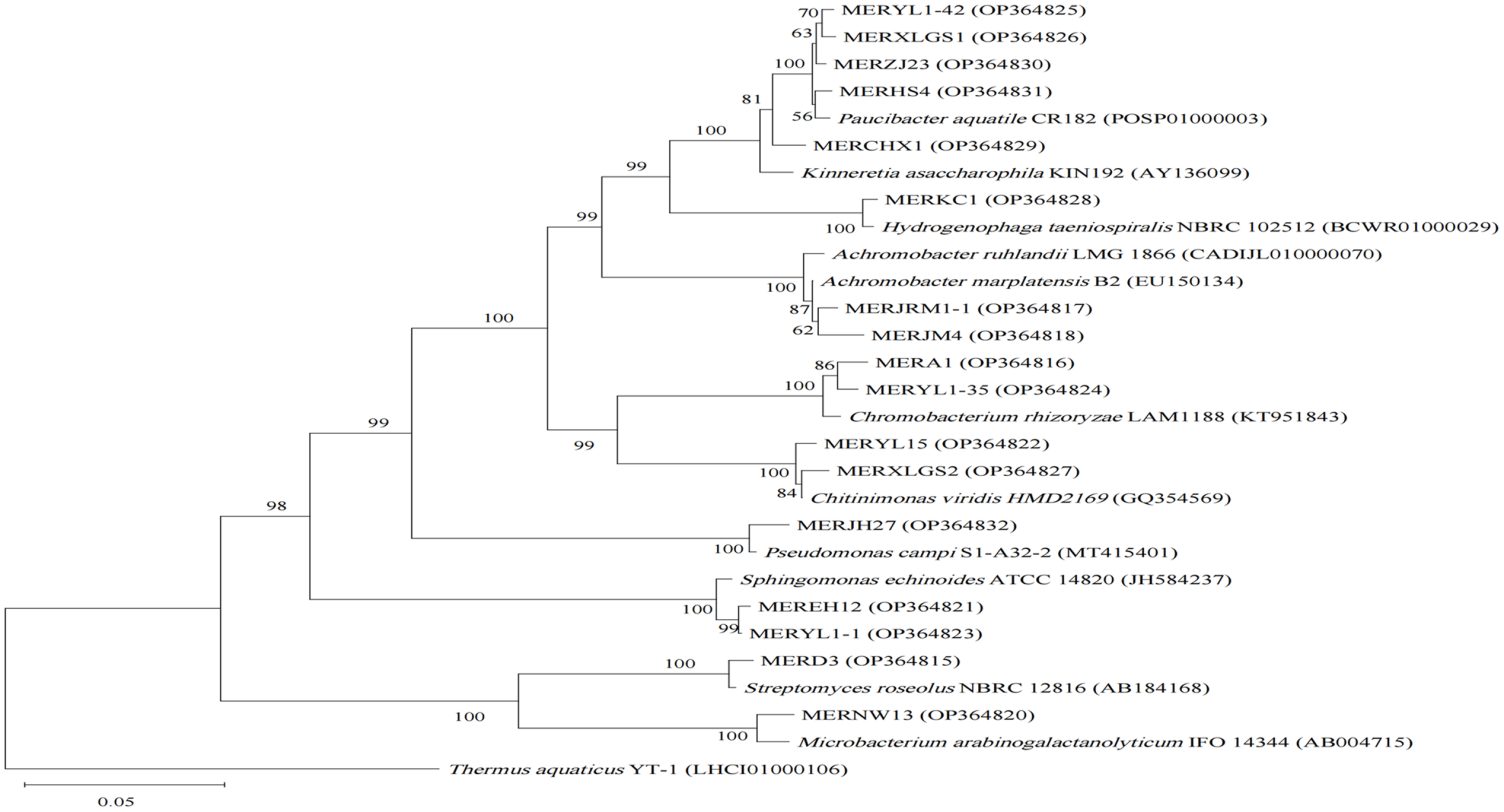
Neighbor-joining phylogenetic tree of 16S rRNA gene sequences of the algicidal bacterial isolates for both cyanobacteria and algae, and their closest relatives. Accession numbers of the isolates (GenBank) and their relatives (EZbiocloud) are shown in parentheses. *Thermus aquaticus* YT-1 was used as outgroupsBootstrap values ≥50% (Bootstrap = 1,000) are shown at the branch nodes. Bar, 0.05 substitutions per nucleotide position.

Comparisons of 16S rRNA gene sequences in EZbiocloud database indicated our 77 isolates are assigned to four bacterial phyla, Proteobacteria (55 strains), Firmicutes (13 strains), Actinobacteria (5 strains), and Bacteroidetes (4 strains) (Table 1; Supplementary Table S2). Generally, *Pseudomonas* and *Bacillus* are the most abundant assigned gram-negative and gram-positive groups, respectively. However, distinctive taxonomic profiles were found among the 3 algicidal bacterial subgroups (Figures 1–3).

For the cyanobacterial algicidal bacterial strains (Figure 1; Supplementary Table S2), Proteobacteria, primarily Betaproteobacteria and Alphaproteobacteria, is the most abundant phylum, with Comamonadaceae being the most abundant family (6 out of 15 Proteobacteria strains). These strains are assigned to a wide range of genus, such as *Asticcacaulis*, *Rhizobium*, *Hydrogenophaga*, and *Paucibacter*. Firmicutes is the 2^nd^ largest phylum with *Bacillus* as the dominant genus (4 out of 7 Firmicutes strains). However, the largest group within the algal algicidal bacterial isolates (Figure 2; Supplementary Table S2) is Gammaproteobacteria, with Pseudomonadaceae and *Pseudomonas* being the dominant family and genus, respectively. This fewer number of gram-positive algicidal bacterial isolates than gram-negative isolates is consistent with previous studies (Meyer et al., 2017). Among them, MERYS24 and MERBGS9 are the new isolates belonging to *Paenibacillus polymyxa*, who exhibited a similar algicidal ability with our previously reported bacterial isolate, *Paenibacillus polymyxa* MEZ6 (Zhao et al., 2022). Interestingly, most bacterial isolates in the broad-target subgroup (Figure 3; Supplementary Table S2) are from Betaproteobacteria, with Comamonadaceae as the dominant family; this pattern is partly similar to that of the cyanobacterial algicidal bacteria. However, some genera like *Chromobacterium* and *Chitinimonas* were only detected in this subgroup. All these phylogenetic differences collectively reflected the high target-specificities of our algicidal bacterial isolates.

Among these 77 bacterial isolates, it is important to note that a number of strains may be newly discovered algicidal bacteria. Literature search suggested that algicidal abilities of more than 10 genera are rarely reported in previous studies (Table 1). For example, MERSZ-4 (*Inhella inkyongensis* IMCC1713) and MERSZ-2 (*Flectobacillus roseus* GFA-11) can inhibit cyanobacteria *Anabaena* sp. PCC 7120 and *Synechocystis* sp. PCC 6803 (Table 1; Supplementary Table S2), respectively. MERC1 (*Massilia eburnean* 10R5-21) showed a strong algicidal ability against alga *C. reinhardtii* 21gr (Table 1; Supplementary Table S2). These isolates may greatly broaden our understanding of the diversity of algicidal bacteria, and provide new materials for future mechanism studies and applications.

### Distributions of bacterial isolates

**Figure 4:**
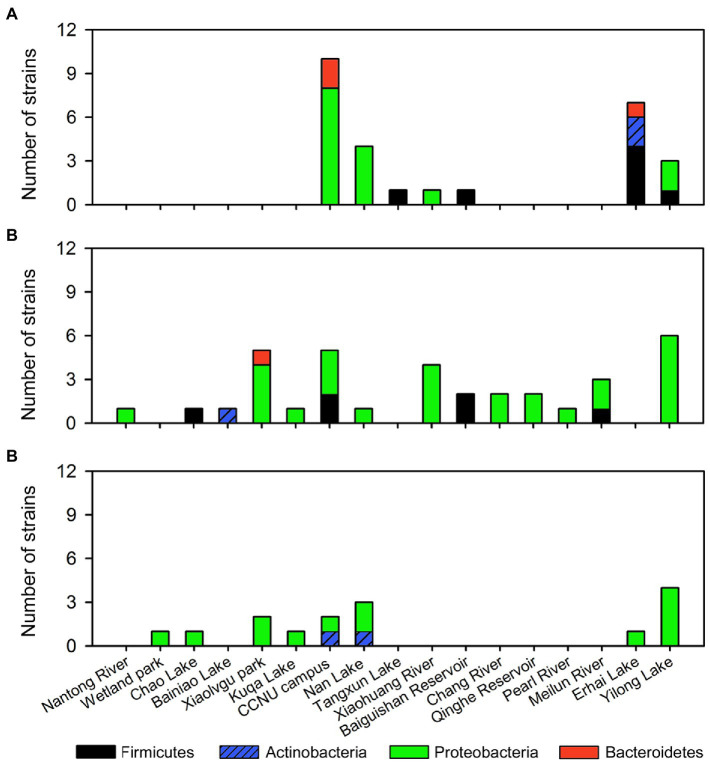
Distribution of screened algicidal bacterial strains. **(A)** Cyanobacterial algicidal bacteria. **(B)** Algal algicidal bacteria. **(C)** Broad-target algicidal bacteria for both cyanobacteria and algae.

Interesting distribution patterns were also found among these isolates (Figure 4). While the strains with the activity against cyanobacteria are mainly from CCNU campus, Nan Lake, Erhai Lake, and Yilong Lake (Figure 4A), strains with the activity against algae are widely distributed across most sampling sites (Figure 4B). CCNU campus, Nan Lake, and Yilong Lake are the only sites where all 3 subgroups of algicidal bacteria (cyanobacterial, algal and the broad-target algicidal bacteria) were isolated (Figures 4A–C). However, there are several sites where only one type of algicidal bacteria was found, such as Meilun river and Qinghe Reservoir with only the algal algicidal bacteria (Figure 4B). Interestingly, most gram-positive bacterial strains (Firmictes and Actinobacteria) for cyanobacteria are from Erhai Lake. And the broad-target algicidal bacteria were only isolated form several sites such as Nan Lake and Yilong Lake (Figure 4C).

Some bacterial strains, such as those assigned to *Paenibacillus polymyxa* ATCC 842, *Pseudomonas hunanensis* LV, and *Sphingomonas echinoides* ATCC 14820, were repeatedly isolated from different samples, indicating they may have a wide geographic distribution in the sampling locations. Most of these kinds of isolates showed similar algicidal patterns. For example, all the *P. hunanensis* LV-assigned strains are grouped into algicidal bacteria. However, certain strains may have distinct algicidal target-specifies, even they are taxonomic-assigned to one species. In the isolates assigned to *Flectobacillus roseus* GFA-11, while MERXLGS3 (from Xiaolvgu park) can effectively inhibit the algal strains, another strain MERSZ-2 (from CCNU campus) is specifically functional against *Synechocystis* sp. PCC 6803. These data highlighted the possible functional diversities of algicidal bacteria within the same species level.

### Representative algicidal strains

**Figure 5:**
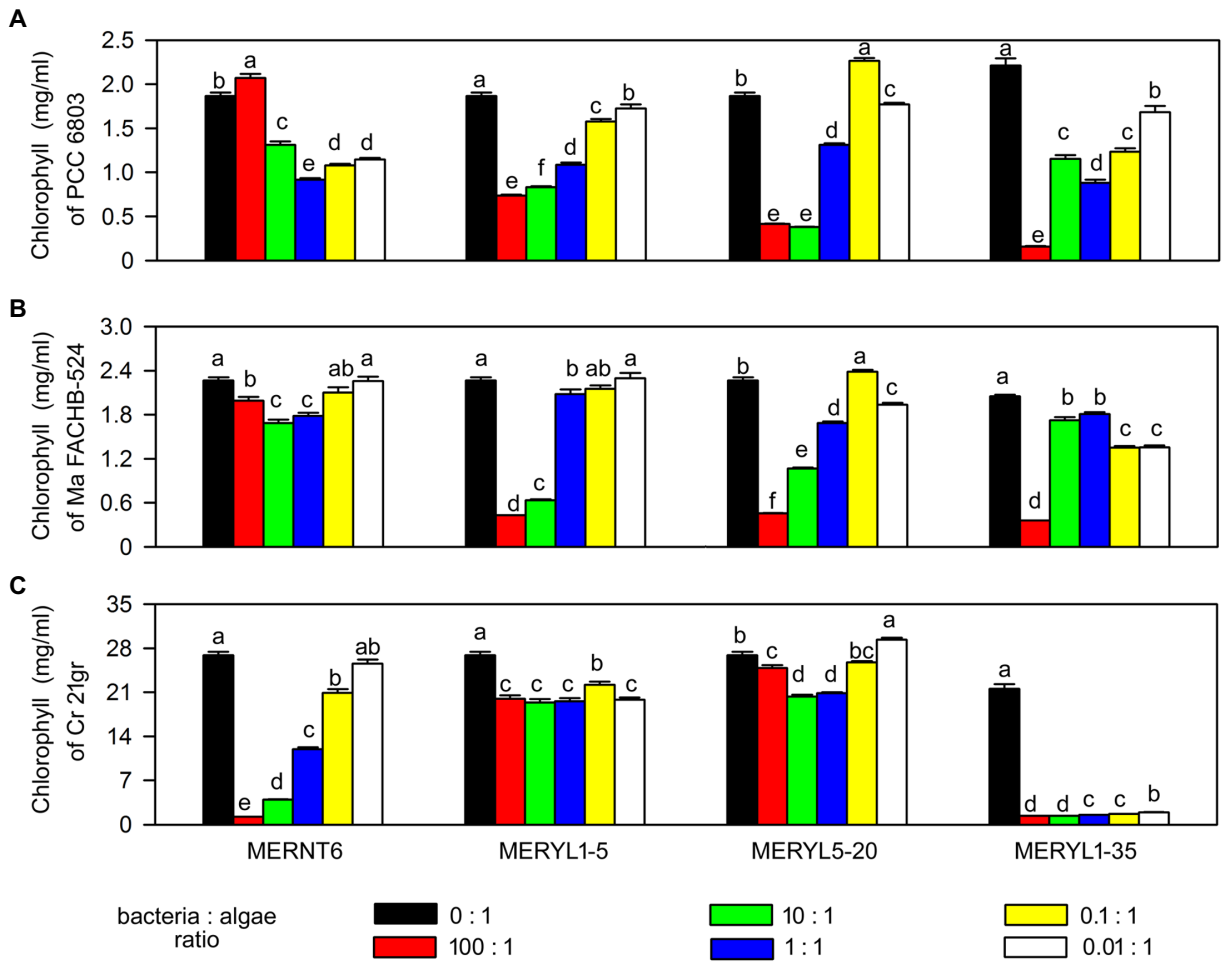
Effects of bacteria: algae inoculum ratios on algicidal abilities of selected bacterial strains. Chlorophyll α contents of *Synechocystis* sp. PCC6803 (PCC 6803) and *M. aeruginosa* FACHB-524 (Ma FACHB-524) were shown in **(A)** and **(B)**, respectively. Total chlorophyll contents of *C. reinhardtii* 21gr (Cr 21gr) were shown in **(C)**. Data are shown as the mean ± SE from three independent assays. Bars marked with the same letter are not significantly different as determined by one way ANOVA and Duncan *post hoc* test (*p* < 0.05).

We selected several strains, including algal algicidal bacterial strain MERNT6, cyanobacterial algicidal bacterial strains MERYL5-20 and MERYL1-5, and the broad-target algicidal bacterial strain MERYL1-35, for more detailed studies. We first tested the effects of bacteria: algae inoculum ratio on algicidal activity. As shown in Figure 5, in most cases the tested strains showed a dose dependent algicidal activity, the higher bacterial inoculum ratio, the stronger algal-inhibiting ability. Notably, MERYL1-35 showed a high algicidal rate (>80%) against *C. reinhardtii* 21gr even the inoculum ratio is below 1:1. However, it required a 100:1 inoculation ratio for a similar strong effect when co-inoculated with *Synechocystis* sp. PCC6803 and *M. aeruginosa* FACHB-524 (comparing Figures 5A–C), reflecting that algicidal ability of the same bacterium can be greatly different when the target is changed. Microscopic observations indicated all these strains can easily induce discoloration of chlorophyll and disruption of cells of the target alga or cyanobacterium (Data not shown).

**Figure 6:**
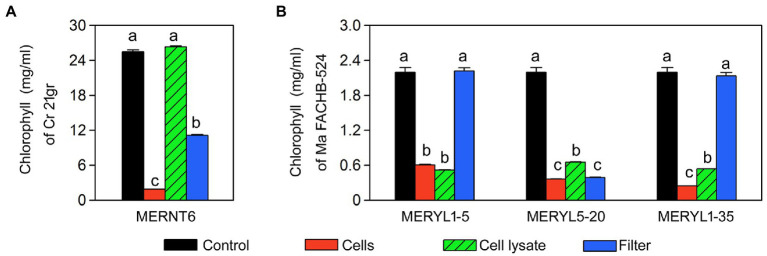
Algicidal abilities of selected bacterial strains in different co-inoculation treatments. Total chlorophyll contents of *C. reinhardtii* 21gr (Cr 21gr) were shown in **(A)** and chlorophyll α contents of *M. aeruginosa* FACHB-524 (Ma FACHB-524) were shown in **(B)**. Control: Alga or cyanobacterium alone; Cells: co-inoculation using living bacterial cells; Cell lysate: co-inoculation using living bacterial cells; Filter: bacterial cells and algal or cyanobacterial cells are separated with filters. Data are shown as the mean ± SE from three independent assays. Bars marked with the same letter are not significantly different as determined by one way ANOVA and Duncan *post hoc* test (p < 0.05).

We further tested whether algicidal abilities of these strains require the living cells and the direct contacts between cells. As shown in Figure 6, all the cell lysates of the strains had a high algal-inhibiting effect, except for that of MERNT6 (Figure 6A), suggesting the living cells of MERNT6 are required for its algicidal ability. In the co-inoculations with filters, only MERYL5-20 and MERNT6 (Figure 6B) showed a strong algicidal activity when they and the target algal cells were separated with a filter, suggesting these bacteria may secret certain algicides that can easily pass through the filter. However, MERYL1-5 and MERYL1-35 may require a direct cell–cell contact to inhibit the target algal strains. All these results suggested algicidal mechanisms can be greatly diverse among our isolated strains.

## Discussion

In the past decades, various algicidal bacteria have been extensively isolated and their studies have greatly improved our understanding of microbial interspecies interactions between phytoplankton and the associated bacteria. However, our current knowledge of algicidal bacteria and their field applications are still limited. Systematic screen, identification and activity comparisons of algicidal bacteria still remain rarely. In this study, we screened algicidal bacterial strains from various freshwater aquatic environments in China using several cyanobacteria and algae as the targets, and evaluated their algal-inhibiting abilities, target-specificities and distributions. All these results should shed new insights into the diversities and distributions of algicidal bacteria in freshwater aquatic environments in China.

Phylogenetic diversities of our isolates re-emphasized the unevenness of algicidal bacterial taxa. These isolates are assigned to Proteobacteria, Firmicutes, Actinobacteria, and Bacteroidetes bacterial phyla, of which Proteobacteria is the most abundant group (Figures 1–3; Supplementary Table S2). This is consistent with the data of previous studies, particularly for algal algicidal bacteria (Meyer et al., 2017; Yang et al., 2020). Among the isolates, notably, the algicidal abilities of a number of strains were identified for the first time to our knowledge (Table 1; Supplementary Table S2). These bacterial strains can provide new resources for future mechanism and application studies. Together with the geographic distributions of our isolates (Figure 4), our results suggested that there are rich algicidal bacterial resources in our sampling sites.

Among these sampling sites, CCNU campus is the site with the highest total tested bacterial number and algicidal isolate number. This is probably due to the convenient transportation and in-time laboratory treatment. However, its percentage of algicidal bacteria is about 5% among the total tested bacteria, which is less than those of Xiaolvgu park and Erhai Lake (Supplementary Table S1). These results imply bacterial screen can be greatly affected by sample source and storage in transportation. These differences among our sampling sites also suggested HABs event and water quality may also greatly affect algicidal bacterial species (Supplementary Table S1). However, since the isolations were only among a few culturable bacteria of the microbial communities and the algicidal ability assays were only conducted with limited target strains, more resource collections and identifications are still needed in future works for a better understanding of algicidal bacteria in Chinese freshwater aquatic environments.

Activities of algicidal bacterial can be cell–cell contact dependent or independent, and may involve various functional compounds. Our isolates may provide new opportunities for the studies of algicidal mechanisms. For example, based on the co-inoculation results, we postulate MERYL1-35 may require a cell–cell contact manner to lyse algal strains, while MERYL5-20 may excrete certain algicidal compounds which can pass though the filter (Figure 6). We believe these different algicidal manners should also be detected in other isolates, and new algicidal compounds may be involved in these interactions. Additionally, based on the target taxa, we have approximately grouped the isolates into cyanobacterial, algal and broad-target algicidal bacteria subgroups (Figures 1–3). This implies some algicidal bacteria may attack the cellular structures or pathways uniquely present in prokaryotic cells and eukaryotic cells, respectively. In fact, greater diversities can be found at the target species level (Table 1; Supplementary Table S2). These specificities can be the results not only of specific metabolic exchanges, molecular recognition and evolutionary history as suggested previously (Seymour et al., 2017; Stock et al., 2019), but also of the cell density, growth phase and environmental condition. For example, the bacterial isolate MERNT6 showed a low activity against *Synechocystis* sp. PCC6803 when a high bacterial inoculum was used (Figure 5A), which is different from the common inoculum dos-dependent inhibitory effect. Illustrating these mechanisms should greatly contribute to our understanding of algicidal bacteria-algae interactions.

Our isolates can also be beneficial to improve the applications of algicidal bacteria. With the strong algal inhibitory effects, algicidal bacteria play roles in composition shaping and functions of aquatic microbial community. However, while algicidal bacteria have been widely identified, their applications in HABs control are still limited (Coyne et al., 2022). Differing from the assays conducted in laboratory conditions, the actual performance of algicidal bacteria can be greatly influenced by various factors when released to environment. First, bacterial survival can be greatly affected by environmental growth conditions (Ramos et al., 2001), such as nutrient and temperature, which can lead to insufficient cell concentrations for inhibiting algal growth. Second, the interactions between microorganisms within a microbial community may be highly complex and the native microorganisms may form negative interactions with the introduced algicidal bacteria, which may significantly attenuate the algal-lysing abilities (Soda et al., 1998; Abreu and Taga, 2016; Wu et al., 2021). In addition, water bloom often consists of multiple phytoplankton species whose abundances may change seasonally (Hedberg et al., 2020), their control may not be easily accomplished by the use of only one type of algicidal bacterial strain. To overcome these limitations, it can be greatly beneficial to identify more new algicidal bacterial strains with enhanced performance. Our current approach can be used in the isolations with the new samples, and facilitate the evaluation of bacterial activities and target-specificities in HABs control. All the algicidal bacterial collections can be further utilized in the selections of specific candidate strains for a given location, and in the combinations with multiple bacterial species for complex HABs control (He et al., 2021). Additionally, the functional algicidal genes or metabolites of these bacterial isolates can be useful for developing efficient algal-inhibiting approaches with genetic manipulations.

In summary, we here isolated bacteria from various freshwater aquatic samples in China and screened a number of algicidal bacterial strains. All these bacterial strains greatly broaden our understanding of the diversity and distribution of algicidal bacteria in China freshwater aquatic environments, and may provide new algicidal microbial resources for future mechanism studies and applications.

## Data availability statement

The datasets presented in this study can be found in online repositories. The names of the repository/repositories and accession number(s) can be found in the article/Supplementary material.

## Author contributions

SR: investigation, resources, methodology, formal analysis, and writing—original draft. YJ and NZ: investigation and methodology. JM and XP: investigation. BX: conceptualization, methodology, validation, supervision, writing—review and editing, and funding acquisition. All authors contributed to the article and approved the submitted version.

## Funding

This work was supported by the National Natural Science Foundation of China (31970109 and 31570098) and the Fundamental Research Funds for the Central Universities (CCNU22JC015 and KJ02502022-0450). We thank Zhengjun Chen for the help in sampling at Yilong Lake.

## Conflict of interest

The authors declare that the research was conducted in the absence of any commercial or financial relationships that could be construed as a potential conflict of interest.

## Publisher’s note

All claims expressed in this article are solely those of the authors and do not necessarily represent those of their affiliated organizations, or those of the publisher, the editors and the reviewers. Any product that may be evaluated in this article, or claim that may be made by its manufacturer, is not guaranteed or endorsed by the publisher.

## Footnotes

^1^ http://chlamycollection.org

^2^ http://algae.ihb.ac.cn
